# Coupling between infraslow activities and high‐frequency oscillations precedes seizure onset

**DOI:** 10.1002/epi4.12425

**Published:** 2020-08-08

**Authors:** Hiroaki Hashimoto, Hui Ming Khoo, Takufumi Yanagisawa, Naoki Tani, Satoru Oshino, Haruhiko Kishima, Masayuki Hirata

**Affiliations:** ^1^ Department of Neurological Diagnosis and Restoration Graduate School of Medicine Osaka University Suita Japan; ^2^ Department of Neurosurgery Otemae Hospital Osaka Japan; ^3^ Endowed Research Department of Clinical Neuroengineering Global Center for Medical Engineering and Informatics Osaka University Suita Japan; ^4^ Department of Neurosurgery Graduate School of Medicine Osaka University Suita Japan

**Keywords:** high‐frequency oscillation, infraslow activity, intracranial electroencephalogram, phase‐amplitude coupling, theta band

## Abstract

Infraslow activities and high‐frequency oscillations (HFOs) are observed in seizure‐onset zones. However, the relation between them remains unclear. In this study, we investigated phase‐amplitude coupling between infraslow phase (0.016‐1 Hz) and HFOs' amplitude of focal impaired awareness seizures followed by focal to bilateral tonic‐clonic seizures, in a 28‐year‐old right‐handed man with a dysembryoplastic neuroepithelial tumor. We recorded five habitual seizures. After the time of seizure onset, a significant increase in the power of HFOs was observed, and the power was significantly coupled with θ (4‐8 Hz) phase. In contrast, coupling of infraslow activities and HFOs surged a few minutes before the seizure‐onset time, and ictal HFOs discharged after that. Collectively, our results show that coupling of infraslow activities and HFOs precedes the seizure‐onset time. We infer that such coupling may be a potential biomarker for seizure prediction.

**Key Points**

- Coupling between infraslow phase and high‐frequency amplitude precedes the seizure‐onset time.

## 1 INTRODUCTION

Wideband data including those from very slow shift to high‐frequency oscillations (HFOs) can be obtained by intracranial electroencephalogram (iEEG). Physiological HFOs are induced by several tasks in healthy participants,^1^ whereas pathological HFOs are observed in epileptic patients.^2^ Direct current (DC) shifts and infraslow activities, which are very slow‐frequency components, are also recorded in relation to epileptic seizures.^3^, ^4^, ^5^ DC shifts, infraslow activities, and HFOs are suggested as useful biomarkers for the detection of the epileptogenic zone.^4^, ^6^, ^7^

The relationship between ictal infraslow activities and HFOs remains unclear. A few reports suggest that ictal infraslow activities and HFOs are located in the same regions^6^ and that ictal infraslow activities precede ictal HFOs.^7^ The relationship between low‐frequency and high‐frequency bands was previously analyzed using the phase‐amplitude coupling (PAC) method.^8^ Ictal HFOs have been shown to be coupled by δ, θ, α, and β.^9^, ^10^, ^11^, ^12^ However, it is unknown how the infraslow phase is coupled with HFOs' amplitude.

In this study, we investigated ictal‐related HFOs using PAC with the infraslow phase (0.016‐1 Hz) in a patient with focal epilepsy because of a dysembryoplastic neuroepithelial tumor (DNT). We hypothesized that coupling between the infraslow phase and the HFOs' amplitude occurs in relation to seizures.

## 2 MATERIALS AND METHODS

The Ethics Committee of Osaka University Hospital approved this case study (no., 19193). Informed consent was obtained by the opt‐out method on our center's website. The study adhered to the Declaration of Helsinki.

A 28‐year‐old right‐handed male patient with no known history of perinatal and developmental problems, nor of febrile seizures, began suffering from seizures at 25 years old. The intractable seizures were focal impaired awareness seizures, characterized by a palpitation aura, followed by motionless staring and automatic mouth movements. These types of seizures were observed approximately once a week and were sometimes followed by a focal to bilateral tonic‐clonic seizure. Interictal scalp EEG recorded spike‐wave complexes over the right temporal region. Furthermore, magnetic resonance imaging (MRI) images showed a cystic lesion in the right mesial temporal lobe (MTL).

**Figure 1:**
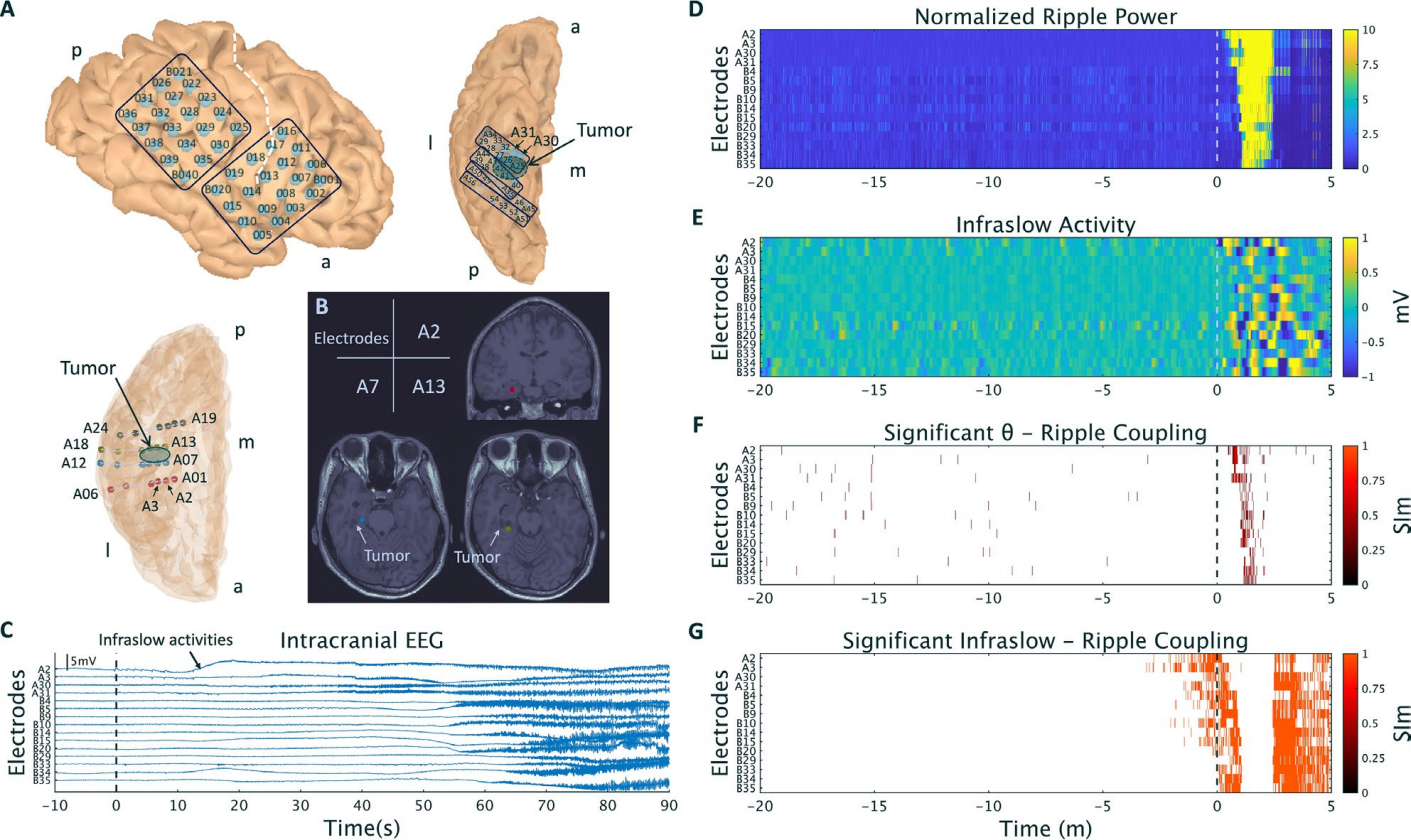
Electrode information and intracranial EEG, ripple power, infraslow activities, and coupling between ripples and θ or infraslow phases at one representative seizure. A, A1‐6, A7‐12, A13‐18, and A19‐24 electrodes, which were depth electrodes, were inserted into the parahippocampal gyrus, the anterior region of the tumor, the posterior region of the tumor, and the lingual gyrus, respectively. a. anterior; p. posterior; m. medial; l. lateral. B, The location of A2, A7, and A13 electrodes is shown on MRI T1WI. The cystic tumor is shown. C, Raw intracranial EEG waveforms. 0 s corresponds to the seizure‐onset time (SOT) determined by a conventional way. In A2, initial infraslow activities were observed. D, The normalized ripple power started to increase after the SOT, from the A2 electrodes (0 s) and propagated to other electrodes. E, Infraslow activities (blue or yellow colored) are observed after the SOT. F, Significant θ‐ripple SIm shows the cluster after the SOT. G, Significant infraslow phase‐ripple SIm is observed even before the SOT. Representative electrodes are shown on the vertical axis.

A total of 96 intracranial electrodes, including grid, strip, and depth type electrodes (Unique Medical Co. Ltd.), were implanted (Figure 1A) in the right hemisphere. A cystic lesion in the right MTL was shown by T1‐weighted MRI (Figure 1B).

Electrode maps were created using FreeSurfer (https://surfer.nmr.mgh.harvard.edu) and Brainstorm (http://neuroimage.usc.edu/brainstorm/) (Figure 1A). Intracranial electroencephalogram signals were measured at a sampling rate of 1 kHz with a 10‐seconds time constant using a 128‐channel digital EEG system (EEG 2000; Nihon Kohden Corporation), and digitized by a 333‐Hz lowpass filter and a 60‐Hz notch filter to eliminate the AC line noise using the BESA Research software (BESA GmbH). The iEEG signals were digitally re‐referenced to a common average of the total implanted electrodes. Four depth electrodes were each inserted into the parahippocampal gyrus, the anterior part of the cystic lesion, the posterior part of the cystic lesion, and the lingual gyrus (Figure 1A, B). Three electrodes (A6, A12, and A18), which were external to the brain, were excluded from the analyses.

Intracranial electroencephalogram signals were analyzed from 20 minutes before to 5 minutes after the seizure‐onset time (SOT). The SOT was determined by visual inspections of iEEG signals. To create HFO power series, we used 80‐250‐Hz band‐pass filtering for ripples and 250‐330‐Hz band‐pass filtering for fast ripples in combination with the Hilbert transform.^13^ The summing of HFO power series in one second was normalized by averaging one‐second HFOs' power in the initial one‐minute. The normalized HFOs' power was calculated every second. To extract the infraslow waveform, we used a 0.016‐1‐Hz band‐pass filter. A band‐pass filter with a two‐way least‐squares finite impulse response filter (pop_eegfiltnew.m from the EEGLAB toolbox, https://sccn.ucsd.edu/eeglab/index.php) was applied to iEEG signals for 60‐minute data. Extra signals were cut down, and subsequently, band‐passed signals corresponding to a total of 25 minutes including seizures were obtained.

For cross‐frequency analysis of HFOs with infraslow, δ (1‐4 Hz), θ (4‐8 Hz), α (8‐13 Hz), and β (13‐30 Hz) bands, we used the synchronization index (SI),^13^ which is a complex number. Therefore, we used the magnitude of SI (SIm), which varies between 0 (completely desynchronized phases) and 1 (perfectly synchronized phase values). We calculated SIm using a 1‐s time window, which sequentially shifted every 33 ms

For the statistical assessment of SIm, the phase time series of HFOs' amplitude was shifted in time by a random amount, and bootstrapped SIm (SImb) was calculated using the lower frequency phase. This procedure was repeated 1000 times to create the distribution of SImb.^13^ At each iteration in the distribution of SImb, the maximum values of SImb were stored. The values at 95% of the distribution of the maximum were a familywise error (FWE)‐corrected threshold, which was applied to the observed SIm for the solution of multiple comparisons.^14^ SIm values over the FWE‐corrected threshold were statistically significant (Figure 1F,G).

**Figure 2:**
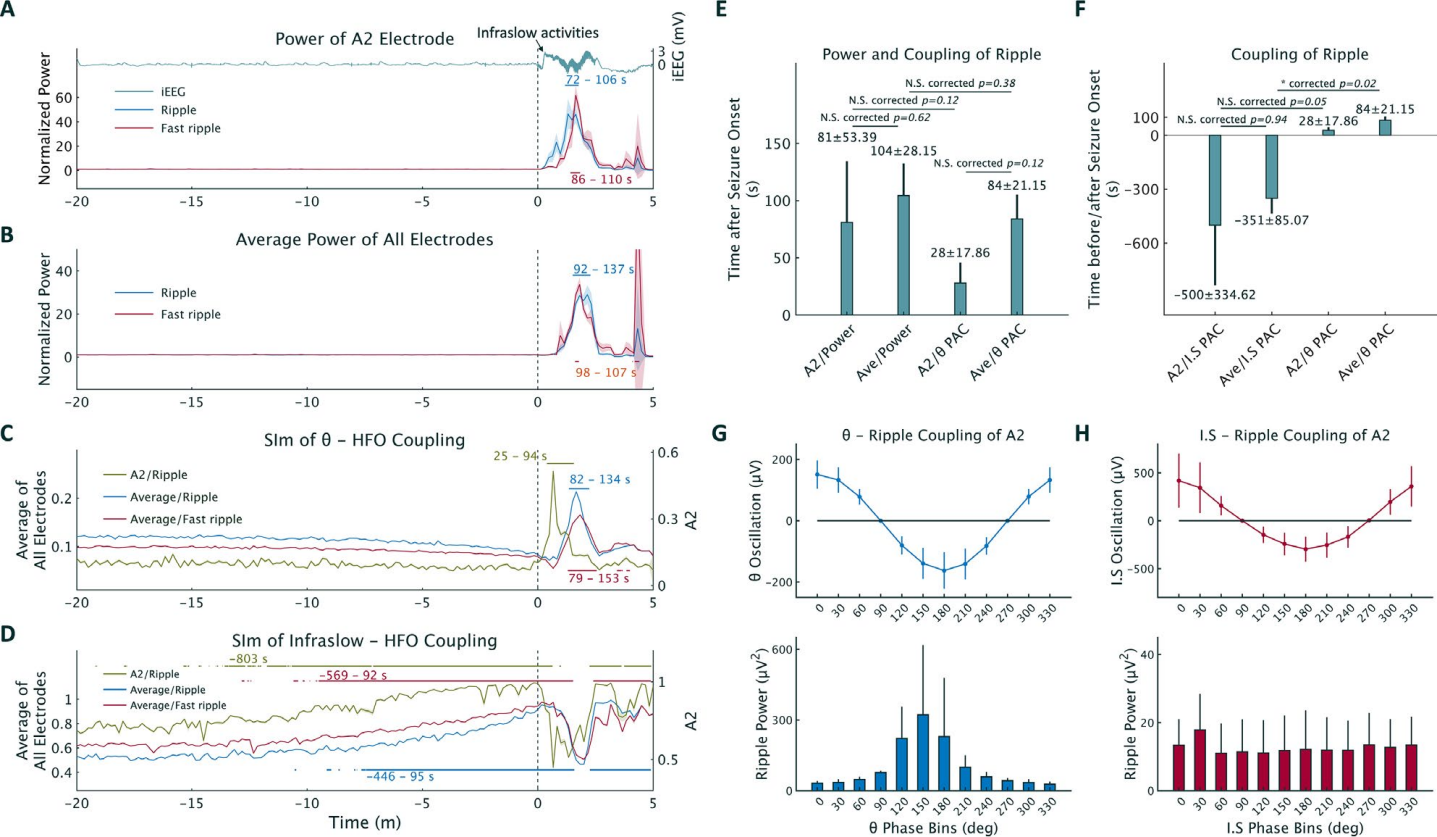
Temporal plots from one representative seizure, which is the same seizure as in Figure 1 (A‐D), and results obtained from the average of all five seizures (E‐H). 0 s corresponds to the seizure‐onset time (SOT). Significant changes are indicated by the horizontal red, green, and blue lines (one‐tailed permutation test with familywise error‐corrected threshold) (A‐D). A, Ripple and fast ripple power recorded from the A2 electrodes were significantly increased after the SOT. The raw intracranial EEG (iEEG) signals from the A2 electrodes showed infraslow activities. B, Averaged ripple and fast ripple power calculated from all electrodes changed significantly after the SOT. C, A significant increase in the θ‐HFOs SIm value recorded from the A2 electrodes or from the average of all electrodes was observed after the SOT. The timing of SIm increase assessed from the A2 electrodes was earlier than that assessed from the average of all electrodes. D, Significant increases in the infraslow‐HFOs' SIm value recorded from the A2 electrodes or the average of all electrodes were observed about 7‐13 min before the SOT. E, The timing when significant changes were observed in terms of power burst and θ‐ripple phase‐amplitude coupling (PAC) was compared for changes detected at the same electrode (A2 or average of all electrodes [“average”]); the times of detecting power bursts and θ‐ripple coupling were also compared between the A2 and the “average” electrodes (single‐sided Wilcoxon signed‐rank test with Bonferroni correction). The timing of θ‐ripple coupling recorded from the A2 electrodes was the earliest compared to the timing of θ‐ripple coupling recorded from the “average” and power bursts recorded from A2 and “average” electrodes. F, The timing when a significant infraslow (I.S) PAC was recorded from the “average” electrodes was compared to that recorded from the A2 electrodes, as well as to the timing of θ PAC also recorded from the “average” electrodes (single‐sided Wilcoxon rank‐sum test with Bonferroni correction). The detection time of infraslow phase‐ripple PAC from the A2 and “average” electrodes was before the SOT. G, During the significant increase in θ phase‐ripple coupling recorded from the A2 electrodes, the trough of θ oscillation was observed at the 180° bin, and the highest ripple power was observed around the trough. H, During the significant increase in infraslow activity‐ripple coupling recorded from the A2 electrodes, the infraslow oscillation showed a trough at the 180° bin, but the ripple power showed no peak. Error bars indicate 95% confidence intervals (CIs) in A‐D and standard deviation in E‐H. 95% CIs in C and D are too narrow to see.

To compare interictal periods with ictal periods of normalized HFOs' power or SIm, we compared the initial 10‐second data, considered to represent an interictal period, with the 10‐second data extracted every second using a permutation test (one‐tailed test). To correct for multiple comparisons, FWE‐corrected threshold (99% threshold) was applied (Figure 2A‐D).

For the phase‐conditioned analysis, the lower frequency phase was divided into 12 intervals of 30° without overlaps from 0° to 330°. At the time when significant SIm values were observed, lower frequency oscillations and ripple power were averaged within each phase interval.

For statistical evaluation, we used single‐sided Wilcoxon signed‐rank test, or single‐sided Wilcoxon rank‐sum test. The problem of multiple comparisons was solved by Bonferroni correction.

## 3 RESULTS

We captured five focal onset impaired awareness seizures involving head turning to the left followed by focal to bilateral tonic‐clonic seizures after the reduction of antiepileptic drugs. The A2 electrodes inserted in the parahippocampal gyrus showed an initial low‐frequency high‐amplitude periodic spiking from 0 seconds (SOT), and subsequently, infraslow activities and low‐voltage fast waves appeared (Figure 1C). The same tendency was observed during all seizures, and we concluded that the seizure onset zone (SOZ) was localized in the right mesial temporal lobe and associated with the cystic legion; infraslow activities are useful to detect the SOZ.^15^ We performed a selective hippocampectomy, also removing the cystic lesion; pathological findings showed that the lesion was DNT. The patient was seizure‐free at the 12‐month follow‐up.

The average maximum SIm was compared among the δ, θ, α, and β bands (single‐sided Wilcoxon signed‐rank test). The θ‐ripple SIm was the highest, but this result was not significant after Bonferroni correction (Figure S1A). Throughout the following analyses, we investigated the SIm of θ‐HFOs.

The results of the same representative seizure are presented in Figure 1C‐G, Figure 2A‐D. After the SOT, increased ripple power was initially observed at the A2 electrodes, spreading to other electrodes (Figure 1D); infraslow oscillations (scaled with color, infraslow activities indicated as yellow or blue [Figure 1E]) and significant θ‐ripple couplings were observed (Figure 1F). On the contrary, significant infraslow‐ripple couplings were observed even before the SOT (Figure 1G). There were no obvious infraslow activities before the SOT (Figure 1E). Furthermore, the raw iEEG signals showed that there were no obvious ictal changes before the SOT and no infraslow activities while observing significant infraslow‐ripple SIm in Figure 1G (Figure S2).

Temporal plots showed that ripple and fast ripple power solely from the A2 electrodes or the average of all electrodes (hereafter, the “average”) significantly increased after the SOT (Figure 2A,B). Moreover, we observed significant θ‐ripple and θ‐fast ripple SIm values after the SOT (Figure 2C), as well as significant infraslow‐ripple and infraslow‐fast ripple SIm values even before the SOT (Figure 2D), using data solely from A2 electrodes or the “average.” Conversely, in a 60‐minute iEEG segment without HFOs, neither θ‐ nor infraslow activity‐ripple coupling was seen (Figure S3).

Significant increases both in the HFOs' power and θ‐HFOs' coupling were observed after all seizures. In contrast, preictal significant coupling of infraslow activities with HFOs was observed only at the A2 electrodes in 3/5 seizures and at the “average” electrodes in 4/5 seizures. The timing when significant values were first observed was not different between ripples and fast ripples in terms of power or coupling (single‐sided Wilcoxon signed‐rank test; Figure S1B‐E). In the following analysis, we solely investigated ripples as representatives of HFOs.

The average time of detecting the first significant values post‐SOT in terms of power bursts did not differ between the A2 and “average” electrodes, while the detection times of power bursts and θ phase coupling were also not different when these were recorded from the “average” electrodes. The timing of θ phase coupling detection at the A2 electrodes was earlier than that of power burst detected at the A2 electrodes, and earlier than the detection time of θ phase coupling at the “average” electrodes; however, significant differences disappeared after Bonferroni correction (single‐sided Wilcoxon signed‐rank test; Figure 2E). The timing when significant values were first observed in terms of infraslow activity‐ripple coupling detected at the A2 or the “average” electrodes was earlier than the SOT. Moreover, infraslow coupling detected at the “average” electrodes occurred significantly earlier than θ phase coupling detected at the “average” electrodes (corrected *P* = .02, single‐sided Wilcoxon rank‐sum test with Bonferroni correction; Figure 2F).

To identify the lower frequency phase in which ripples were coupled during significant coupling, the average lower frequency oscillation and ripple power were calculated from the A2 electrodes. Oscillations both of θ and infraslow phases showed a trough at 180° (upper rows in Figure 2G, H). The obvious peak of ripple power was observed at 150° of the θ phase; however, the ripple power tuned by the infraslow phase showed no peak (bottom rows in Figure 2G, H).

## 4 DISCUSSION

The main finding of this study is that by using the PAC method, we showed significant infraslow activity‐HFO coupling a few minutes before seizures onset. This occurred before any significant increase in HFO power. The coupling between 0.1‐Hz frequency and HFOs was previously reported^9^; however, our study reveals the coupling between the 0.016‐1‐Hz frequency band, representing infraslow activities and HFOs. Previous studies have demonstrated that HFOs are coupled with δ, θ, or α bands after seizures,^9^, ^10^, ^11^, ^16^ which is consistent with our results, especially for the θ band. In this study, the earliest that the θ phase‐HFOs' coupling was detected by the A2 electrodes inserted into the SOZ occurred after the seizures. In contrast, coupling of infraslow activities and HFOs began to increase before seizures, and this surge was followed by HFOs' discharge.

The PAC between the α phase and the power of the HFOs was reported to occur before the increase in power of motor‐related physiological HFOs.^17^ The α‐HFO coupling disappeared, and then, the power of the HFOs increased. Therefore, a hold‐and‐release model of the HFOs' power was proposed, indicating that a strong coupling restricted the activities of the HFOs and an attenuation of the coupling released the activities of the HFOs. In this study, the infraslow activity‐HFOs coupling occurred before the HFOs discharge. Our findings suggest that the surge of infraslow phase‐HFOs' amplitude coupling may represent a neural process for the preparation of HFOs' discharge. Moreover, we investigated five focal to bilateral tonic‐clonic seizures, and we showed that HFOs' discharge propagated from the SOZ electrodes (A2) to other electrodes. Therefore, we inferred that both significant changes of HFOs' power and infraslow‐HFOs coupling could be obtained from the average of all electrodes.

We could not detect obvious infraslow activities with conventional methods before the SOT. The conventional method for infraslow activity detection involves visual identification^7^; therefore, we inferred that PAC methods enabled detection of changes which could not have been detected visually.

Our results showed that, after seizures, the θ‐HFOs coupling was the highest compared to HFOs' coupling with other lower frequency bands and that this coupling detected by A2 electrodes inserted into the SOZ appeared earlier than the HFOs' power burst. These results suggest that θ phase‐ripple coupling is a potential biomarker for seizure detection, but not for seizure prediction. However, since infraslow‐HFOs coupling was significantly high even a few minutes before seizures, we infer that it may be used as a potential biomarker for seizure prediction.

This study has some limitations. First, our results were obtained from one patient with focal to bilateral tonic‐clonic seizure due to a brain tumor. It remains unclear whether the same results can be obtained in patients with neocortical epilepsy. More seizures must be analyzed to confirm whether infraslow activity‐HFOs' coupling is a useful biomarker for seizure prediction. Simulation methods are a useful way to provide scientific evidence of infraslow activity‐HFO coupling before seizures. Second, all seizures were captured after the reduction of antiepileptic drugs, and thus, they might not represent usual seizures. Finally, fast ripples were obtained by 250‐330‐Hz band‐pass filtering because our sampling rate was 1 kHz. However, fast ripples usually correspond to 250‐500/600 Hz. Considering this limitation, our results might show no difference between ripples and fast ripples.

## CONFLICT OF INTEREST

None of the authors has any conflict of interest to disclose. We confirm that we have read the Journal's position on issues involved in ethical publication and affirm that this report is consistent with those guidelines.

## AUTHOR CONTRIBUTIONS

HH conceived the study, collected the data, created MATLAB‐based program, analyzes the data, created all the figures, and was primarily responsible for writing the manuscript. MH and HMK performed the surgery. All authors took clinical care and evaluated the patients. HK and MH supervised this study. All authors reviewed the manuscript.

## ETHICAL APPROVAL

We confirm that we have read the Journal's position on issues involved in ethical publication and affirm that this report is consistent with these guidelines.

## Supporting information

Supplementary Material
