# Supplementary material for: Coupling between infraslow activities and high‐frequency oscillations precedes seizure onset
Source: Epilepsia Open. 2020 Aug 8;5(3):501–6. doi: 10.1002/epi4.12425 (PMC7469835; doi:10.1002/epi4.12425)
Supplement: Supplementary file 1 — Supplementary Material [file EPI4-5-501-s001.docx]

Supplementary Information for

**Coupling between Infraslow activities and High-Frequency Oscillations precedes Seizure Onset**

Hiroaki Hashimoto*, Hui Ming Khoo, Takufumi Yanagisawa, Naoki Tani, Satoru Oshino, Haruhiko Kishima, Masayuki Hirata

*Hiroaki Hashimoto, M.D., Ph.D.

Email: [yousyou0720@gmail.com](mailto:yousyou0720@gmail.com)

FIGURE S1 Comparison of the maximum SIm values of ripple coupling among lower-frequency bands and comparison of the average time calculated from five habitual seizures when the first significant differences were observed. A, The SIm value of θ phase-ripple coupling was highest among other lower-frequency bands (single-sided Wilcoxon signed-rank test with Bonferroni correction). B, At the A2 electrodes, the timing of significant ripple power increases did not differ from that of fast ripple (single-sided Wilcoxon signed-rank test). C, There was no significant difference between the average ripple and fast ripple power (single-sided Wilcoxon signed-rank test). D, In θ phase coupling, there was no significant difference between ripples and fast ripples (single-sided Wilcoxon signed-rank test). E, In infraslow phase coupling, there was no significant difference between ripples and fast ripples (single-sided Wilcoxon signed-rank test). N.S., not significant.

FIGURE S2 Raw ECoG signal of all electrodes. Electrodes numbers are shown on the vertical axis. Total 60 s data are shown in one page, and minutes before or after seizure onset time (SOT) are also indicated in the parentheses on the horizontal axis. The time of 0 min corresponds to the SOT. Initial infraslow activities after the SOT are first observed in the A2 electrodes (red arrow). Before the SOT, there are no obvious ictal changes. During the time from about three minutes before the SOT to the SOT when the significant infraslow - ripple coupling were observed in Figure 1G, there are no clear infraslow changes.

FIGURE S3 Representative interictal results of total 60 minutes ECoG data. The horizontal axis corresponds to the time (min). A, The normalized ripple (80 – 250 Hz) power are indicated as color. The vertical axis corresponds to each electrode, and the order of the electrodes are same to Figure S2. There is no ictal ripple burst. B, The temporal plot of normalized rippled power calculated from the A2 electrodes or the average are indicated as blue or red line in respectively. Ictal ripple activities are not observed. C, The temporal plot of θ-ripple or infraslow-ripple SIm values from the average of all electrodes are shown as blue or red line. SIm increases are not observed in either of θ- and infraslow-ripple coupling.
